# Intravitreal Avastin for Choroidal Neovascularization Associated with Stargardt-Like Retinal Abnormalities in Pseudoxanthoma Elasticum

**DOI:** 10.4103/0974-9233.71586

**Published:** 2010

**Authors:** Giuseppe Querques, Anna V. Bux, Francesco Prascina, Nicola Delle Noci

**Affiliations:** 1 Department of Ophthalmology, Policlinico Riuniti di Foggia, University of Foggia, Foggia, Italy; 2 Department of Ophthalmology, Centre Hospitalier Intercommunal de Creteil, University Paris XII, Creteil, France

**Keywords:** Avastin, Intravitreal Bevacizumab, Pseudoxanthoma Elasticum, Stargardt’s Disease

## Abstract

The aim of the study was to describe a patient with pseudoxanthoma elasticum (PXE), showing Stargardt-like retinal abnormalities, who underwent treatment with intravitreal bevacizumab for subfoveal choroidal neovascularization (CNV) of the right eye (RE). A 57-year-old woman with diagnosis of angioid streaks, retinal flecks, and chorioretinal Stargardt-like atrophy due to PXE was referred to our department for sudden decreased vision in her RE (20/160). Upon a complete ophthalmologic examination, including fluorescein angiography (FA), and optical coherence tomography (OCT), the patient was diagnosed with subfoveal CNV of the RE. Owing to the subfoveal localization of the CNV, the patient was submitted to intravitreal bevacizumab injection. At the 1-month follow-up, visual acuity (VA) improved (20/40), and FA and OCT revealed the CNV closure. Twelve months after the treatment, the patient’s VA remained stable with no recurrence of active CNV. On the basis of our findings, a single intravitreal bevacizumab injection seems to induce total regression of CNV complicating PXE, in a patient showing Stargardt-like retinal abnormalities. Further investigations are required to confirm our results.

## INTRODUCTION

Pseudoxanthoma elasticum (PXE) is a hereditary systemic disease of the connective tissue characterized by progressive calcification, fragmentation, and degeneration of elastic fibers in the skin, eye, and cardiovascular system. The gene defect in PXE has been characterized as a loss of function mutation in the adenosine triphosphate-binding cassette subtype C number 6 gene (ABCC6).^1^ Modification in the extracellular matrix, which is important in the health and vitality of the retinal pigment epithelium (RPE), appears to be related this mutation. The most common ocular findings are angioid streaks, which are irregular, tapering linear breaks in Bruch’s membrane that typically emanate from the optic disk. Other ocular features associated with PXE include peau d’orange, optic nerve drusen, retinal crystalline bodies, focal atrophic pigment epithelial lesions, and pattern dystrophy of the macula.^2^–^6^

We present an interventional case report describing a patient with PXE, showing Stargardt-like retinal abnormalities,^6^ who underwent treatment with intravitreal bevacizumab (Avastin, Genentech, Inc., South San Francisco, CA, USA) for subfoveal choroidal neovascularization (CNV).

## CASE REPORT

**Figure 1:**
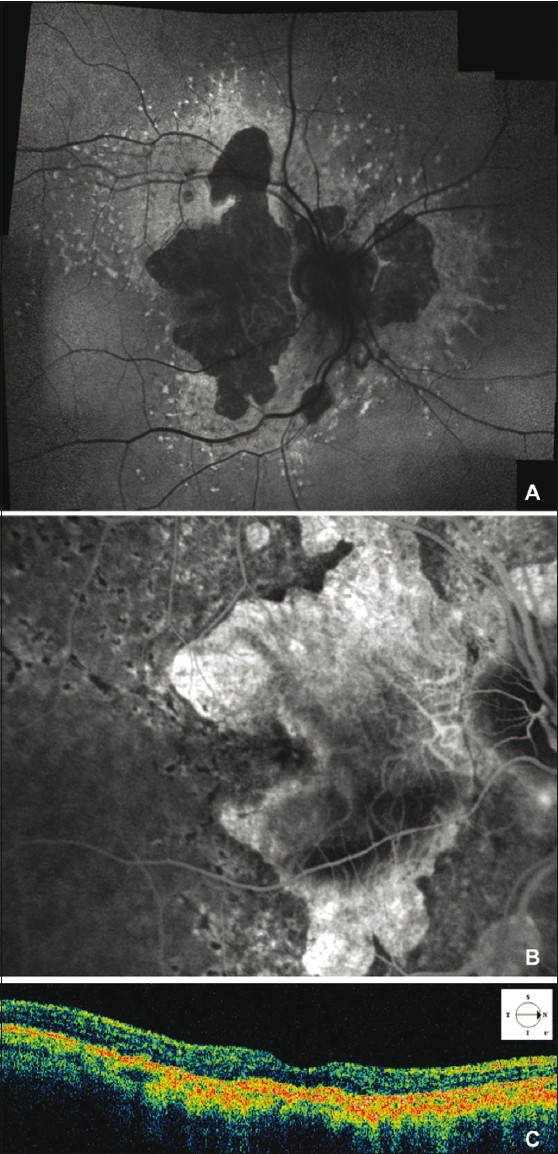
Fundus autofluorescence shows retinal flecks and areas of welldefined retinal atrophy (A). Late phase fluorescein angiography of the RE shows macular leakage form subfoveal choroidal eovascularization (CNV), associated with cystoid macular edema (CME), within a large area of well-defined atrophy (B). The hypofluorescence of the retinal flecks, is still discernable despite the hyperfluorescent background due to retinal pigment epithelium changes. Optical coherence tomography scan demonstrates moderately reflective mass corresponding to the subfoveal CNV, associated with shallow neurosensory retina elevation and CME in the macular area (C)

**Figure 2:**
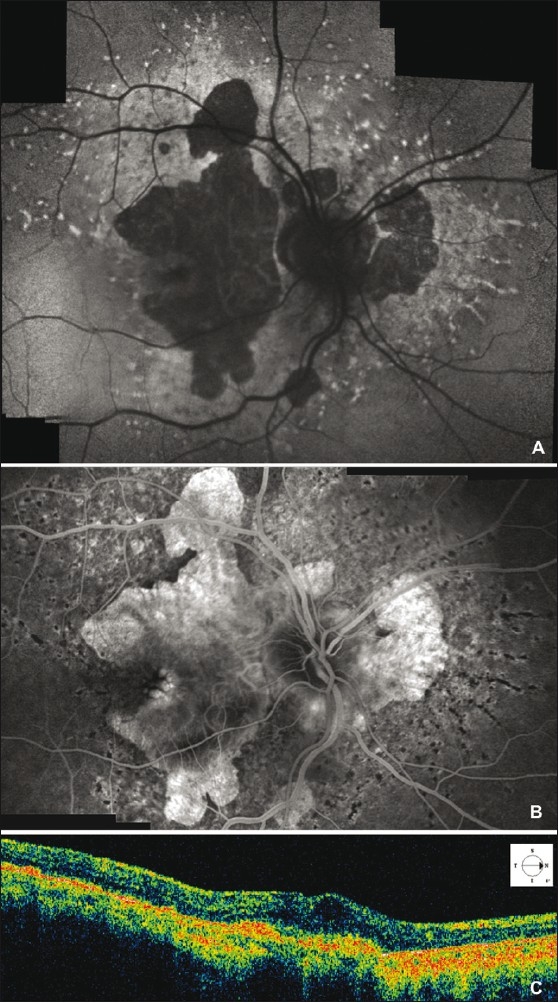
Fundus autofluorescence shows retinal flecks and areas of well-defined retinal atrophy 1 month after intravitreal bevacizumab injection (A). Fluorescein angiography late frame of the right eye, one month after intravitreal bevacizumab injection, shows moderate heterogeneous hyperfluorescence and absence of leakage due to the choroidal neovascularization (CNV) closure (B). Optical coherence tomography scan demonstrates high reflective mass corresponding to the subfoveal fibrotic CNV, and absence of both neurosensory retina elevation and cystoid macular edema in the macular area (C)

A 57-year-old woman diagnosed with angioid streaks, retinal flecks and chorio-retinal Stargardt-like atrophy due to PXE (homozygous nucleotide substitution in exon 9 of the ABCC6 gene [C1132T] introducing a stop codon at position 378 [Q378X]) was referred to our department for sudden decreased vision in her right eye (RE). The patient signed a comprehensive consent form according to Good Clinical Practice guidelines, before proceeding with all examinations and treatments. Her best-corrected visual acuity (BCVA) was 20/160 in the RE and 20/400 in the left eye (LE). On fundus biomicroscopy, the macula of the RE showed a subfoveal greenish-gray lesion associated with cystoid macular edema (CME), and the macula of the LE showed a very large fibro-atrophic lesion. Fluorescein angiography (FA) and optical coherence tomography (OCT-3, Humphrey-Zeiss, San Leandro, CA, USA) of the RE revealed subfoveal CNV associated with CME [Figure 1]. After discussing treatment options, including the option of intravitreal bevacizumab, the patient requested intravitreal bevacizumab for treatment. Intravitreal bevacizumab (1.25 mg/0.05 mL) was administered without complication. At the one-month follow-up, BCVA of the RE improved to 20/40, FA revealed closure of CNV and OCT revealed total resolution of the associated CME [Figure 2]. Twelve months after treatment, the patient’s BCVA remained 20/40 without recurrence of either active CNV or CME.

## DISCUSSION

Pattern dystrophy of the macula is a rare ocular feature associated with PXE.^2^–^6^ The most common ocular findings in PXE are angioid streaks, and subfoveal CNV is the major cause of vision loss associated with angioid streaks.^2^ Here we report a patient with PXE, with Stargardt-like retinal abnormalities,^6^ who successfully underwent treatment with intravitreal bevacizumab for subfoveal CNV. Although CNV formation is not a rare finding in cases of angioid streaks due to PXE, this case is unique because PXE presents with retinal flecks and areas of well-defined retinal atrophy typical of Stargardt’s disease. Hence, we describe this case as “Stargardt-like” retinal abnormalities.

Based on Karacorlu *et al*.’s observations,^7^ we elected not to perform photodynamic therapy (PDT) with verteporfin in our patient because of the risk of disciform transformation of the neovascular process. Given that ABCC6 mutation seems to be responsible for modification of the extracellular matrix, which is important in the health and vitality of the RPE, we believe that in the era of anti-vascular endothelial growth factor therapies, one should avoid treat such patients with PDT, which could cause further damage to the genetically compromised RPE cells.

Our case is unusual in that, CNV complicating PXE and angioid streaks, did occur in a patient showing Stargardt-like retinal abnormalities. In this patient, intravitreal bevacizumab showed a long-lasting effect (12 months), as evaluated by BCVA, FA, and OCT.

On the basis of our findings, just one intravitreal bevacizumab injection seems to induce total regression of CNV complicating PXE and angioid streaks, in a patient showing Stargardt-like retinal abnormalities, within 1-month after treatment. Further study is required to confirm our results.
